# Understanding how false memories relate to daily life in Alzheimer’s disease and related dementias

**DOI:** 10.1002/alz.092625

**Published:** 2025-01-03

**Authors:** Emily Waskow, Renee DeCaro, Brenna Hagan, Noundy Mazile, Anna Marin, Myna Chadalavada, Kristina Morreale, Meltem Karaca, Katherine W. Turk, Andrew E. Budson

**Affiliations:** ^1^ VA Boston Healthcare System, Jamaica Plain, MA USA

## Abstract

**Background:**

In Alzheimer’s disease and related dementias (ADRD), memory deficits as assessed through neuropsychological testing and self‐report are often defined by instances of forgetting. However, the presence of false memories (e.g., falsely remembering actions or events) may also be prominent, with the potential to carry significant implications for an individual’s quality of life. The goal of this study is to quantify these false memories and their impact on daily functioning and independence in ADRD.

**Method:**

We tested older adult Veterans (26 male, 4 female; ages 62‐92, *M* = 75, *SD* = 6.5) from a Memory Disorders Clinic to assess false memories, as reported by an informant, and their correspondence with the patients’ 1) outcomes from neuropsychological testing, 2) self‐report measures (e.g., depression, anxiety, quality of life, and independence in activities of daily living), 3) performance on experimental paradigms, and 4) event‐related potentials (ERPs). In preliminary analysis (N = 30), we computed correlations between false memories and outcomes. Structural equation modeling will be undertaken with the complete sample (N = 75) to investigate the relationships between false memories and other relevant experimental and clinical outcomes, including latent factors underlying neuropsychological evaluation and outcomes related to ERPs (e.g., N400).

**Result:**

Consistent with previous research (Turk et al., 2020), false memories were significantly and positively related to instances of forgetting, *r*(29) = 0.93, *p* < .01. We further demonstrated that instances of false memories were inversely related to education, *r*(29) = ‐0.41, *p* < .05, inversely related to scores on the MoCA, *r*(29) = ‐0.55, *p* < .01, and positively related to functional activities, *r*(29) = 0.75, *p* < .01. These initial results demonstrate that greater instances of false memories were associated with lower reported educational attainment, lower performance on a cognitive screener, and greater dependence in functional daily living activities.

**Conclusion:**

We demonstrated that false memories occur just as frequently as forgetting, supporting previous research. Additionally, we found that independence in activities of daily living is linked with greater instances of false memories. Final analyses will determine the relationship between false memories and other health indicators, with implications for how false memories impact patient’s daily lives.

